# Editorial Notes

**Published:** 1907-12

**Authors:** 


### Editorial Notes.

---

**Bristol Royal Infirmary.**

A special meeting of the Governors of this institution was held in the board-room on December 5th, to consider changes in the rule 36, when, as the outcome of the recent controversy and in accordance with the agreement arrived at previously between the Committee and the Faculty, the following rule was put to the meeting and carried:—

> "No member of the Honorary Staff shall hold any Union or Club appointment. No member of the full Staff, or of the Assistant Staff, shall hold any other General Hospital appointment or more than one special Hospital appointment, but this rule shall not preclude members of the Honorary Staff accepting purely consulting and not active appointments at another hospital. That the full Physicians shall limit their practice to medical work. That the full Surgeons shall limit their practice to surgical work. That each of the Specialists shall limit his practice to his speciality,"

thereby annulling the rule proposed and carried at the last Board meeting, and to which the Medical Faculty could not give its consent.

**Food-fads and Faddists.**

Dr. W. Hale White, in his address in Medicine, delivered at the Annual Meeting of the British Medical Association at Exeter, pleading for accuracy of thought in medicine, and speaking on the habit of using the term irregular gout as a cloak for pathological ignorance, remarked that "the symptoms of irregular gout were commonly ascribed to an excess of uric acid or bodies allied to it in the blood. Some do this without knowing the amount of uric acid present in the blood of a healthy person, without drawing any distinctions between endogenous and exogenous uric acid, without any attempt to estimate the uric acid in the patient's blood, without any experimental evidence that the injection of uric acid into the blood produces the symptoms in question, and without any thought of the fact that in some forms of leukæmia there is an excess of uric acid in the blood without the symptoms supposed to indicate suppressed gout. This doctrine has become so widespread, that even the public tell us that their symptoms are caused by an excess of acid. Having assumed, not proved, that the symptoms the patient has are due to irregular gout, and having assumed, not proved, that this is due to an excess of uric acid, the next assumption made is that certain foods cause an excess of uric acid; some say carbohydrates, some say fats, some say proteids, and it would be quite easy for a patient to consult three doctors in turn, and if he followed all, his diet would be water and nothing else. He who believes proteids harmful is the most artistic, for he has an eye to colour, and may, as a concession, allow white meat although he prohibits red. Where on earth is the justification for this? Are there any experiments showing that steak leads to more uric acid in the blood than chicken? Have a hundred cases of so-called irregular gout been published and contrasted with another hundred similar in their treatment, except that in one series red meat was replaced by an equal amount of white? Surely you will agree with me that all this is not a credit to us as members of a scientific profession. The simple fact that although gout has become much less common the consumption of meat has enormously increased, ought alone to make those who forbid proteids pause. The imagination of some has carried them still further. The fact that both gout and chronic osteo-arthritis are long-lasting diseases of joints has led them to think that as sufferers from one should be dieted, so those afflicted with the other; hence we find patients who have chronic osteo-arthritis forbidden various articles of food, sometimes, for example, sugar. Looked at calmly this is extraordinary, for there is not an atom of evidence that any particular article of food influences chronic osteo-arthritis. Have we in some of our statements about the harmfulness of particular foods advanced beyond Ibu Haukal, who in about A.D. 950, describing the inhabitants of Palermo, said, "Their evil habit of eating raw onions in excess; for there is not a person among them, high or low, who does not eat them in his house daily, both in the morning and evening. This is what has ruined their intelligence, and affected their brains, and degraded their senses, and distracted their faculties, and crushed their spirits, and spoiled their complexions, and so altogether changed their temperament that everything, or almost everything, appears to them quite different from what it is." I strongly suspect that in those days onions were a cause of irregular gout.

The literary output on the subject of foods and food-fads has of late been enormous. The older views of Banting, the more modern ones of Fletching, the grape-nuts of Bernard Shaw, the apples of Haddow, the various vegetable fancies of Eustace Miles, the cheese diet of Dr. Haig, the use of Bulgarian sour milk, and that of a crowd of patent foods and drinks of various kinds, lead to the natural conclusion that a diet grounded on common sense is, perhaps, after all the best. Some are afraid of meat because of its toxins, and of broths because of their purin and waste products; others fear that a vegetable diet may end in gastric dilatation, and that salads may harbour uncooked microzoa and bacteria; milk and butter must be dangerous inasmuch as they harbour the germs of tubercle; even bread may charge the blood with acids, whilst water may contain the germs of typhoid, and alcohol is a concentrated poison: hence it may be assumed that the only reasonable course is to eat, as appetite prompts, those viands which have been found by the experience of centuries to be harmless, and that neither the use of meat, soup, wine, condiments, fruits, vegetables, or coffee will be likely to shorten our days, although the abuse of some of them may.

It has been pointed out by Pawlow that we cannot live on chemical formulæ or on calories, and Chittenden maintains that the dietetic customs and habits of mankind are not much to the point, inasmuch as instinct and craving are not wise guides. What has been called the "cabbage inspired" writings of the vegetarians are still less to the point, but direct physiological experiment should be the only scientific guide to an "ideal diet." Chittenden pleads that over-feeding is the predominant dietetic sin; his newer experiments on dogs on the effects of a low proteid dietary confirm his former views that the commonly accepted "normal" and "standard" diets contain an excessive amount of proteid, and he has shown by observations on men of the Army Hospital Corps, and more recently again on university athletes, that the body weight and nitrogen equilibrium can be maintained on an amount of proteid food far below the usual dietary standards.[1]

Far more important than the regulation of the quality of the food appears to be the important questions relating to the quantity of the same. These are discussed at considerable length by Van Noorden[2] in chapters on under-feeding and over-feeding. The general conclusion appears to be that it is impossible to draw up fixed, rigid rules for the individual; the personal equation is the all-important question, hence there is much excuse for the numerous vagaries in diet now in vogue, and much scope still remains to the manufacturing chemist for the unlimited increase of eccentricities in food and drink.

The importance of the personal element in dietetics is well illustrated by Dr. Leonard G. Guthrie.[3] He writes as follows:—

"The diet suitable for neurotic children is indeed always a matter of perplexity—no fixed rules for all are possible. Some abhor milk in any form, and would live on meat and pickles only; to others, meat and fat in almost any shape are abominable; some can only relish bread and butter, sweets and jam; some can hardly be induced to touch fresh vegetables or fruit, others would live on nothing else; some regard with suspicion the slightest variation and innovation in their diet, others will not eat the same food twice." He further adds that

[1] *The Nutrition of Man.* Chittenden. Heinemann, 1907.

[2] *Metabolism.* Translated by Walker Hall. Heinemann, 1907.

[3] *Functional Nervous Disorders in Childhood.* Oxford University Press, 1907, p. 25.

"our tastes and distastes with regard to food are generally formed in early life, and what we do not like is almost certain to disagree with us." We all realise how difficult the problems of diet in sickness become when the patient has abhorred milk in early life, and will have none of it afterwards.

**Winsley Sanatorium.**

The second annual report of this Institution has been before us for some months. The medical profession generally is fully alive to the value of the work done in an Institution which has now shown itself to be a necessity, and to be an indispensable auxiliary to the National Association for the Prevention of Consumption. We think, however, that the public has not yet fully realised what good work is being done for the three Counties of Gloucester, Somerset and Wilts, and the City and County of Bristol. The Sanatorium is saving and prolonging many valuable lives, and is doing a great educational work. It is not, however, sufficiently known that whilst its finances remain in the unsatisfactory state which still continues it has been arranged by the Board of Governors that "In case any maintained bed shall not have been actually allocated to a local authority, public body, firm, group of persons, or private donor, who shall have agreed to maintain the same, the committee of management shall be at liberty to admit patients to such bed at the following charges: To persons resident within the Counties of Somerset, Gloucester and Wilts, and the City and County of Bristol, £1 15s. per week; to persons not so resident, £2 10s. per week." During the past year many paying patients have been admitted under this rule, and it is of great assistance to the funds to have these beds fully occupied. Medical men are asked to remember that there is commonly no difficulty in securing admission to any patient on this basis.

We are glad to take this opportunity to welcome the arrival of the newly-appointed medical officer, Dr. A. Lewthwaite, who comes from Dr. Otto Walther's Sanatorium in the Badischer Schwarzwald.

**An Imperial Dispensatory.**

We wish to direct especial attention to a review of the *British Pharmaceutical Codex* for the use of medical practitioners and pharmacists which will be found on page 358. This *Codex* has been published by the authority of the Council of the Pharmaceutical Society of Great Britain, and although it has not quite the authority of the British Pharmacopœia it is likely to be a far more useful work. Every medical man should possess it, and it should be a constant companion both to prescriber and dispenser. We are indebted to Mr. A. L. Taylor, of the Bristol Royal Infirmary, for the excellent summary of a book which must make a great difference in the work of the pharmaceutical dispenser throughout the world.

**"Codex" Review.**

A striking, if unintentional, testimony to the value of the new *Codex* is provided by a little red pamphlet, received apparently by all the members of the medical profession in Bristol, and presumably other places, entitled "A Curious Codex." The noble and disinterested zeal with which the author expresses his righteous indignation at the omission of the mention of "Gingament" will surely be recognised by all prescribers of "Oids."

Two salient facts stand out in happy contrast to the mass of puerility and diluted sarcasm provided by the writer. One, already dealt with in the review of the *Codex* in this number, the need for some distinguishing mark of the B.P. article as compared with B.P.C. The other—the writer's own statement, born doubtless of inward conviction—that his pamphlet is too long.

**University College, Bristol, Faculty of Medicine.**

The Right Hon. Lewis Fry again presided at the annual distribution of prizes to the students of the medical department of the College at the commencement of the medical session in October, when Professor Francis Gotch, M.A., D.Sc., F.R.S., gave a highly stimulating address on the question of University extension, and asked the question: "When will Bristol do what Liverpool has done, and attain its educational manhood." Prof. Gotch, as a Bristolian, is anxious for the reputation of the city. It was his hope and his firm belief that he might very shortly have the great pleasure of rejoicing with his fellow-Bristolians over the realisation of the long-expected University scheme, for when once started on a wide basis, it would astonish them by its vitality and free growth, and would become the pride of those who established and supported it.

Another noteworthy address given recently is that of Prof. Francis Francis on "The Nitrogen of the Atmosphere," particularly in relation to food supply.

The annual dinner was presided over by an old and distinguished student of the College, Mr. J. H. Parsons, F.R.C.S., who was supported by Prof. Francis Gotch as the guest of the evening. Prof. Walker Hall, in responding to the toast of the Bristol Medical School, remarked that this Faculty is a source of strength to the city and county, that it is an important factor in the everyday life of the community, and that it is doing good work, which will at one time or another benefit the individual citizen.

Prof. Gotch's missionary effort on behalf of the University scheme should do much to stimulate the local influences which are working steadily in that direction.

